# Comparison of Trifecta and Pentafecta Outcomes across 3 Surgical Modalities of Partial Nephrectomy (PN) – Open, Lap, and Robotic

**DOI:** 10.15586/jkcvhl.v11i3.308

**Published:** 2024-08-07

**Authors:** Hiranya Deka, N. Mallikarjunarao Medam, Ginil Kumar P., Vishnu P., Manav Gideon, Achuth Ajith Kumar, Yensani Prashanth Reddy, Shivraj Barath Kumar

**Affiliations:** Department of UroOncology, Amrita Institute of Medical Sciences, Kochi, Kerala, India

**Keywords:** Partial Nephrectomy, Trifecta, Pentafecta, Laparoscopic PN, Robotic PN

## Abstract

Renal cell carcinoma (RCC) is the most common solid tumor in the kidney (90%), accounting for about 3% of all cancers in adults. Partial nephrectomy (PN) is the surgical procedure primarily used for the treatment of localized kidney tumors. Two commonly used terms to describe the complexity and success of a partial nephrectomy procedure are “trifecta” and “pentafecta.” Trifecta is defined as Warm ischemia time (WIT) ≤ 25min or Cold ischemia time (CIT) ≤ 60min, Negative surgical margin (NSM), and no perioperative Clavien-Dindo complications (CDC) of Gr 3 or more [8], whereas pentafecta is defined as trifecta plus >90% preservation of e-Glomerular filtration rate (GFR) and no increase in chronic kidney disease (CKD) stage at 12-months post-operative period. We retrospectively analyzed all patients who underwent partial nephrectomy at a single high-volume tertiary centre, from 2012 to 2020. We included patients who underwent partial nephrectomy by any of the three routes including open (OPN), laparoscopic (LPN), or robotic-assisted (RPN), and in which the follow-up data was available. We compared the trifecta and pentafecta outcomes across the three surgical modalities. We had a total of 183 patients in our study. Twenty-nine percent (53 patients) underwent open surgery, 12.6% (23 patients) underwent laparoscopic surgery and 58.5% (107) underwent robotic assisted surgery. The number of patients who fell under the low risk category in the RENAL scoring system were 70(38.3%), intermediate risk 79 (43.2%) and high risk 34 (18.6%). In the high risk RENAL score group, trifecta was achieved in 5 (50%) patients in OPN, 1(50%) in LPN and 7(31.8%) in RPN with no statistically significant difference (p = 0.581) whereas pentafecta was achieved in 3 (30%) patients in OPN, 1 (50%) in LPN and 7 (31.8%) in RPN with no statistically significant difference (0.855). In the overall cohort, mean WIT, mean hospital stay and mean EBL were higher in OPN as compared to LPN and RPN which was statistically significant (p < 0.001), whereas there was no statistical difference in mean operative time between the three modalities (p = 0.580). Renal tumors can be safely treated by RPN or LPN with lesser morbidity as compared to OPN. Trifecta and Pentafecta outcomes had no significant difference among OPN, LPN, and RPN. RPN and LPN may be considered feasible and safe surgical approaches ensuring good functional outcomes.

## Introduction

Renal cell carcinoma (RCC) is the most common solid tumor in the kidney (90%), accounting for about 3% of all cancers in adults. With the increasing prevalence of co-morbidities like diabetes, and hypertension, people are more prevalent and predisposed to worsening renal parameters. RCC is 10–20 times more common in these patients with deranged renal parameters. 4%–8% of RCC occurs as familial, syndromic tumors and these tumors are often bilateral and multiple.

Partial nephrectomy (PN) is recommended to achieve cancer control with renal function preservation, to benefit patients considering all these factors. Partial nephrectomy (PN)/nephron-sparing surgery (NSS) indication has increased due to the significant detrimental effect of radical nephrectomy on renal functions and survival (1,2). Partial nephrectomy may be done by open, laparoscopy, or robotic approaches – based on patient factors, instrument, and skill availability. Open PN has comparable oncologic outcomes with radical nephrectomy (3).

Comparison between laparoscopic and open approaches give similar oncologic outcomes and renal functions (4,5). Laparoscopic and robotic also have comparable outcomes (6,7).

The success of PN is evaluated by two parameters defined as trifecta and pentafecta in recent studies which define short-term and long-term success, respectively. Trifecta is defined as warm ischemia time (WIT) <=25 min or cold ischemia time (CIT) <=60 min, negative surgical margin (NSM), and no perioperative Clavien-Dindo complications (CDC) of grade 3 or more (8), whereas pentafecta is defined as trifecta plus >90% preservation of e-GFR and no increase in chronic kidney disease (CKD) stage at 12 months post-operative period (9).

Here, we compare the trifecta and pentafecta outcomes with RENAL scoring across the three modalities of PN which can give a better understanding to apply them in the future.

## Materials and Methods

We retrospectively analyzed 200 patients of PN done in a single institute from 2012 to 2020 after institutional review board clearance. We have excluded five patients, converted to radical nephrectomy intraoperatively, and 12 patients, done by unclamped technique in open PN. Finally, we got 183 patients who underwent PN by one of the three surgical modalities.

We have collected the data as preoperative patient and tumor characteristics like age, sex, RENAL nephrometry score with its detail, serum creatinine, intraoperative WIT/CIT, estimated blood loss (EBL), and post-operative histopathology with type, grading, margin positivity, CDC, hospital stay, and serum creatinine at 12 months postoperatively. RENAL nephrometry scoring was divided into low (4–6), intermediate (7–9), and high (10–12) risk groups. We compare trifecta (no prolonged ischemia, negative surgical margin, and no CDC of grade 3 or more) and pentafecta (trifecta plus >90% preservation of renal function and no CKD stage upgradation at 12 months post-operative period) outcome across the three surgical modalities according to the RENAL scoring; mean EBL, hospital stay, WIT/CIT, and operative time in the overall cohort is compared across the three surgical modalities separately.

### *Statistical analysis*

Statistical analysis was done using IBM SPSS 20 (SPSS Inc, Chicago, USA). For all the continuous variables, the results are given in mean ± SD, and for categorical variables as percentages. To test the statistically significant association of parameters with the type of surgery, a Chi square test was applied. To test the statistically significant comparison of mean WIT/CIT, mean hospital stay, mean blood loss, and mean operative time among the three surgical modalities in the overall cohort, one-way ANOVA was applied for parametric data. Multiple comparison tests were done by using the Bonferroni test. A p-value < 0.05 was considered statistically significant.

## Results

The total no of cases in our study is 183, with 154 male and 29 female patients who underwent PN, 53 (29%) had open, 23 (12.6%) had a lap, and 107 (58.5%) had robotic. Patient and tumor characteristics are shown in Table 1. Patients with a low RENAL score was 70 (38.3%), an intermediate RENAL score was 79 (43.2%), and a high RENAL score was 34 (18.6%). 112 (61.2%) patients had pathological stage T1a. 147 (80.3%) had clear cells and 91 (49.7%) had Fuhrman grade 2 RCC in the overall cohort (Table 1).

**Table 1:** Patient and tumor characteristics.

	Open	Lap	Robotic
No. of patients (183)	53	23	107
Male (154)	46	19	89
Female (29)	7	4	18
Renal score			
Low (70) (38.3%)	16	16	38
Intermediate (79) (43.2%)	27	5	47
High (34) (18.6%)	10	2	22
Histopathology			
T1a	32	13	67
T1b	12	6	23
T2a	3	0	3
T2b	0	0	0
T3a	3	1	2
Type of tumor			
Clear cell	45	18	84
Papillary	5	2	7
Chromophobe	0	0	4
AML	0	2	4
Oncocytoma	2	1	5
Other	1	0	3
Median creatine			
Preop	1.075	1.14	0.97
Post Op	1.075	1.21	1.03
At 12 months	1.04	1.03	1.09
Avg size of the tumor (cm)	4.6	3.0	3.6

In the low RENAL score group, the trifecta was achieved in 15 (93.8%) patients in OPN, 16 (100%) in LPN, and 36 (94.7%) in RPN with no statistically significant difference (p=0.620), whereas pentafecta was achieved in 15 (93.8%) patients in OPN, 16 (100%) in LPN, and 35 (92.1%) in RPN with no statistically significant difference (0.519).

In the intermediate RENAL score group, the trifecta was achieved in 24 (88.9%) patients in OPN, 2 (40%) in LPN, and 25 (53.2%) in RPN with statistically significant difference (p=0.004), whereas pentafecta was achieved in 20 (74.1%) patients in OPN, 2 (40%) in LPN, and 25 (53.2%) in RPN with no statistically significant difference (0.139) (Table 2).

**Table 2:** Intermediate RS group.

Variables (no. of patients)	Open (27)	Lap (5)	Robotic (47)	p-value
No PI (%)	24 (88.9)	2 (40)	25 (53.2)	0.004
No CDC >=3 (%)	26 (96.3)	5 (100)	47 (100)	0.377
No PM (%)	26 (96.3)	4 (80)	47 (100)	0.023
>90% eGFR preserved (%)	20 (74.1)	4 (80)	25 (53.2)	0.142
No stage change in CKD (%)	21 (77.8)	4 (80)	28 (59.6)	0.226
Trifecta achieved (%)	24 (88.9)	2 (40)	25 (53.2)	0.004
Pentafecta achieved (%)	20 (74.1)	2 (40)	25 (53.2)	0.139

In the high RENAL score group, the trifecta was achieved in 5 (50%) patients in OPN, 1 (50%) in LPN, and 7 (31.8%) in RPN with no statistically significant difference (p=0.581), whereas pentafecta was achieved in 3 (30%) patients in OPN, 1 (50%) in LPN, and 7 (31.8%) in RPN with no statistically significant difference (0.855) (Table 3).

**Table 3:** High RS group.

Variables (no. of patients)	Open (10)	Lap (2)	Robotic (22)	p-value
No PI (%)	5 (50)	1 (50)	7 (31.8)	0.581
No CDC >=3 (%)	7 (70)	1 (50)	19 (86.4)	0.325
No PM (%)	8 (80)	2 (100)	16 (72.7)	0.652
>90% eGFR preserved (%)	3 (30)	1 (50)	8 (36.4)	0.851
No stage change in CKD (%)	3 (30)	1 (50)	9 (40.9)	0.790
Trifecta achieved (%)	5 (50)	1 (50)	7 (31.8)	0.581
Pentafecta achieved (%)	3 (30)	1 (50)	7 (31.8)	0.855

In the overall cohort, mean WIT/CIT, mean hospital stay, and mean estimated blood loss were higher in open PN compared to lap and robotic PN which is statistically significant (p<0.001), whereas there is no statistical difference in mean operative time between the three modalities (p=0.580) (Table 4).

**Table 4:** Comparison between three surgical modalities.

Overall cohort	Open	Lap	Robotic	p-value
Mean WIT/CIT (min)	40.38	25.26	26.59	<0.001
Mean hospital stay (days)	7.98	6.57	6.02	<0.001
Mean estimated blood loss (mL)	392.26	160.0	183.50	<0.001
Mean operative time (min)	221.51	208.18	224.67	0.580

## Discussion

Partial nephrectomy is recommended to achieve cancer control with renal function preservation. Partial Nephrectomy (PN)/nephron-sparing surgery (NSS) indication has increased due to the significant detrimental effect of radical nephrectomy on renal functions and survival. PN may be done by open, laparoscopy, or robotic approaches –, based on patient factors, instrument, and skill availability. Open partial nephrectomy has comparable oncologic outcomes with radical nephrectomy (3). Comparison between laparoscopic and open approaches give similar oncologic outcomes and renal functions (4,5). Laparoscopic and robotic also have comparable outcomes.

Our study is unique in that no study in the past had a comparison of trifecta and pentafecta outcomes in low RS, intermediate RS, and high RS groups with three modalities such as OPN, LPN, and RPN.

In our study, 183 patients (154 were male and 29 were female) underwent PN, 53 (29%) had open, 23 (12.6%) had a lap, and 107 (58.5%) had robotic. Patients with a low RENAL score was 70 (38.3%), an intermediate RENAL score was 79 (43.2%), and a high RENAL score was 34 (18.6%). Histopathology showed clear cell carcinoma – 147 (80.3%), papillary carcinoma – 14 (7.6%), Chromophobe – 4, AML – 6, Oncocytoma – 8, and other types - 4. T stage showed T1a –112 (61.2 %), T1b – 41 (22.4%), T2a – 6, and T3a – 6. Grade of tumor: Gr1 – 39, Gr2 – 96, Gr3 – 45, and Gr4 – 3.

In our study “trifecta” was achieved in 83% OPN, 82.6% LPN, and 63.5% RPN and “pentafecta” was achieved in 71.6% OPN, 82.6% LPN, and 62.6% RPN. In a study by Ghavimi et al. (10), out of 1708 total patients, 746 underwent OPN, 678 LPN, and 284 RPN for a T1 renal mass, “trifecta” was achieved in 53% OPN, 52% LPN, and 47% RPN.

In the intermediate RENAL score group, the trifecta was achieved in 24 (88.9%) patients in OPN, 2 (40%) in LPN, and 25 (53.2%) in RPN with statistically significant difference (p=0.004) which was in the initial learning curve of robotic surgery.

**Figure 1:**
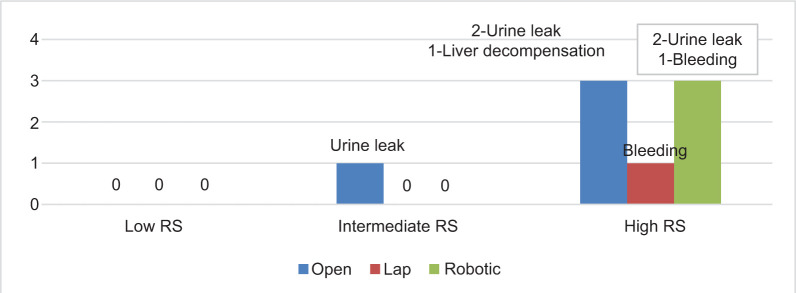
CDC Grade > 3 or more.

In our study, CDC grade 3 or more occurred in one patient in the intermediate RS group – OPN (urinoma undergone DJS), and in the high RS Group, OPN (1 – liver decompensation, 2 – urinoma undergone DJS), LPN (1 – post-op bleeding requiring angioembolization), RPN (2 – urine leaks undergone DJS, 1 – post-op bleeding requiring angioembolization) (Figure 1).

Two single institutional studies by Wang et al. (11) and Kural et al. (12) suggested no difference in blood loss, complications, and margin status, but WIT was lower with RPN. Contradictory to the prior mentioned studies, Haber et al. compared 75 RPN with 75 LPN and noted no significant difference in WIT (18.2 min in RPN vs. 20.3 min in LPN) (13). There was significantly higher blood loss in RPN (323 mL) versus LPN (222 mL). In our study, blood loss in RPN was 183.5 mL and LPN was 160 mL. To summarize, RPNs and LPNs have the same perioperative morbidity in the literature. However, these studies do not account for the location of the tumor or its complexity.

Comparison of OPN to RPN is limited in the literature. Several studies have compared OPN to LPN. Gill et al. compared 1028 OPN with 771 LPN and showed WIT for OPN as 30.7 min versus LPN as 20.1 min (14). In our study, WIT in OPN was 40.38 min and LPN was 25.26 min. In contrast to our study, blood loss was similar for OPN (300 mL) and LPN (376 mL). Pempangkosol et al. compared 58 OPN (median size 2.9 cm) with 85 LPN (2.4 cm) (15), the overall complications were 25% in OPN and 7.8% in LPN.

Lucas et al. did a matched comparison in terms of tumor configuration with the help of nephrometry score, tumor size, age, and gender and compared 54 OPN, 15 LPN, and 27 RAPN (16). There was a similar distribution of low, medium, and high complex lesions in OPN and RPN while LPN had no high complexity lesions. OPN had less operative time and ischemia time but increased blood loss. Postoperatively all groups had lower urine leaks (3.7% RPN, 0 LPN, and 5.6% OPN), with similar GFR. The tumor at the specimen margin was also low (3.7% RPN, 0 LPN, and 7.4% OPN).

In our study, OPN, LPN, and RPN groups had similar median operative time (221.5, 208.18, and 224.67 min) while mean WIT (40.38, 25.26, and 26.59 min), mean hospital stay (7.98, 6.57, and 6.02), and mean estimated blood loss (183.30 mL, 160.0 mL, 392.26 mL) was higher in open PN compared to lap and robotic PN (Table 3).

## Limitations of the Study

Small sample size in each of the groups and selection bias.

## Conclusions

Renal tumors can be safely treated by RPN or LPN with lesser morbidity as compared to OPN. Trifecta and pentafecta outcomes had no significant difference among OPN, LPN, and RPN. RPN and LPN may be considered feasible and safe surgical approaches ensuring good functional outcomes.
